# Breaking the silence: communication failures are the leading errors identified in 10-years of trauma morbidity and mortality conferences

**DOI:** 10.1007/s00068-025-02913-8

**Published:** 2025-06-27

**Authors:** Severin Gloor, Marcel Quante, Beat Lehmann, Beat Schnüriger

**Affiliations:** 1 Department of Visceral Surgery and Medicine, Inselspital, Bern University Hospital, University of Bern, Freiburgstrasse, Bern, 3010 Switzerland; 2 Department of Emergency Medicine, Inselspital, Bern University Hospital, University of Bern, Freiburgstrasse, Bern, Switzerland

**Keywords:** Wounds and injuries, Morbidity, Mortality, Medical errors, Education

## Abstract

**Background:**

Despite global efforts to reduce trauma-related morbidity and mortality, diagnostic and therapeutic errors occur due to the urgency and complexity of care. Trauma morbidity and mortality (Trauma M&M) conferences aim to identify and address such errors to improve clinical outcomes. While most existing data on Trauma M&M conferences originate from the US, insights from Western Europe remain scarce.

**Methods:**

This single-center, retrospective case series analyzed trauma patients discussed at the monthly Trauma M&M at a Swiss Level I trauma center over 10 years (2013–2022). Data were collected from Trauma M&M records and electronic medical charts, including demographics, injury characteristics, timelines, and documented errors. A novel error categorization system was developed, encompassing communication, skill/knowledge deficits, delays, missed injuries, and deviations from algorithms.

**Results:**

Out of 198 trauma cases reviewed, 189 with complete data were further analyzed. The median Injury Severity Score was 32 (IQR 25–43). Of these patients, 77% died within 90 days. A total of 130 potential errors were identified, with communication errors (*n* = 29) being the most frequent, followed by skill/knowledge deficits (*n* = 24) and procedural delays (*n* = 19). Communication errors often triggered subsequent errors, such as missed diagnoses, under-triage, and deviations from protocols. Discussions during the Trauma M&M led to the introduction or refinement of clinical algorithms, including updates to triage protocols.

**Conclusion:**

Communication errors emerged as the leading cause of errors in trauma care, highlighting the need for focused communication training. The Trauma M&M serves as an essential platform for interdisciplinary collaboration, quality improvement, and education. Future research should explore the ripple effects of communication errors and evaluate targeted interventions to optimize trauma care systems.

## Background

Trauma is currently the most common cause of death in persons under 45 years of age and is responsible for the second most patient-years-of-life-lost in Switzerland [1, 2]. Despite the efforts to decrease morbidity and mortality after severe trauma, it still meets the criteria for a global pandemic [3, 4]. Victims of severe trauma are particularly susceptible to errors due to the time-sensitiveness of decisions often in combination with uncertainties about the mechanism of injury or past medical history. Trauma morbidity and mortality conferences (Trauma M&M) aim to identify medical errors and implement corrective actions that may enhance trauma care quality. The vast majority of data on the impact or potential benefits of Trauma M&M originates from the US or non-European countries and focus on potentially preventable deaths [5–7]. Little is known about the implementation and the impact of Trauma M&M from Western European countries. Particularly morbidity seems to be underestimated and underreported [8, 9].

Several categorization systems have been evaluated to classify postoperative complications, quality management and potential errors [10–12]. However, for trauma patients, most of these categorizations are not applicable due to the multidisciplinary setting of trauma care and the high heterogeneity of injured patients. Therefore, an individualized approach and comparative error culture might be helpful [13].

This study aimed to summarize the experience with Trauma M&M and to develop a more comprehensive and generalizable error categorization. The ultimate goal was to identify opportunities for future improvement and seamlessly integrate them into daily practice.

## Methods

### Study site

Bern University Hospital is an academic Level I trauma center in Switzerland, managing approximately 2,500 trauma admissions annually, including 650 patients with severe injuries defined by an Injury Severity Score (ISS) ≥ 16 or head AIS > 2. Initial management follows Advanced Trauma Life Support (ATLS^®^) principles. There are a massive transfusion protocol, a dedicated computed tomography (CT) scanner, and 24/7 availability of all surgical and interventional specialties. Trauma teams are formed based on prehospital information using a tiered system (A- B- or C-trauma teams). In cases of hemodynamic instability, the “C-trauma team” is activated, comprising of an attending emergency physician as team leader, an anesthesiologist and their team, and abdominal, thoracic, and orthopedic surgeons.

### Patient inclusion criteria

This study is a single-center retrospective case series, including all patients discussed during the monthly Trauma M&M at Bern University Hospital, Switzerland. Study period was from January 2013 to December 2022. Of note, any caregiver at Bern University Hospital may schedule patients for discussion at the Trauma M&M.

### Data collection and statistical analysis

Data extracted from the written Trauma M&M records included age, gender, date and time of injury, mechanism of injury, mortality, morbidity, prehospital times, primary survey findings and discussed points. Additionally, patient and injury characteristics were collected from archived electronic medical charts, including prehospital documentation, trauma bay records, surgical, intensive care unit, as well as discharge reports, imaging studies and laboratory results. Moreover, all data were merged with the data entered in the Swiss Trauma Registry including injury characteristics and hemodynamics on admission [14, 15]. Patients with incomplete data were excluded from analysis. Quantitative and qualitative variables were expressed as median (interquartile range) and frequency (percentage). Statistical analysis was performed using SPSS version 28.0.1.1 (IBM, Armonk, New York, USA).

### Study procedure

**Fig. 1:**
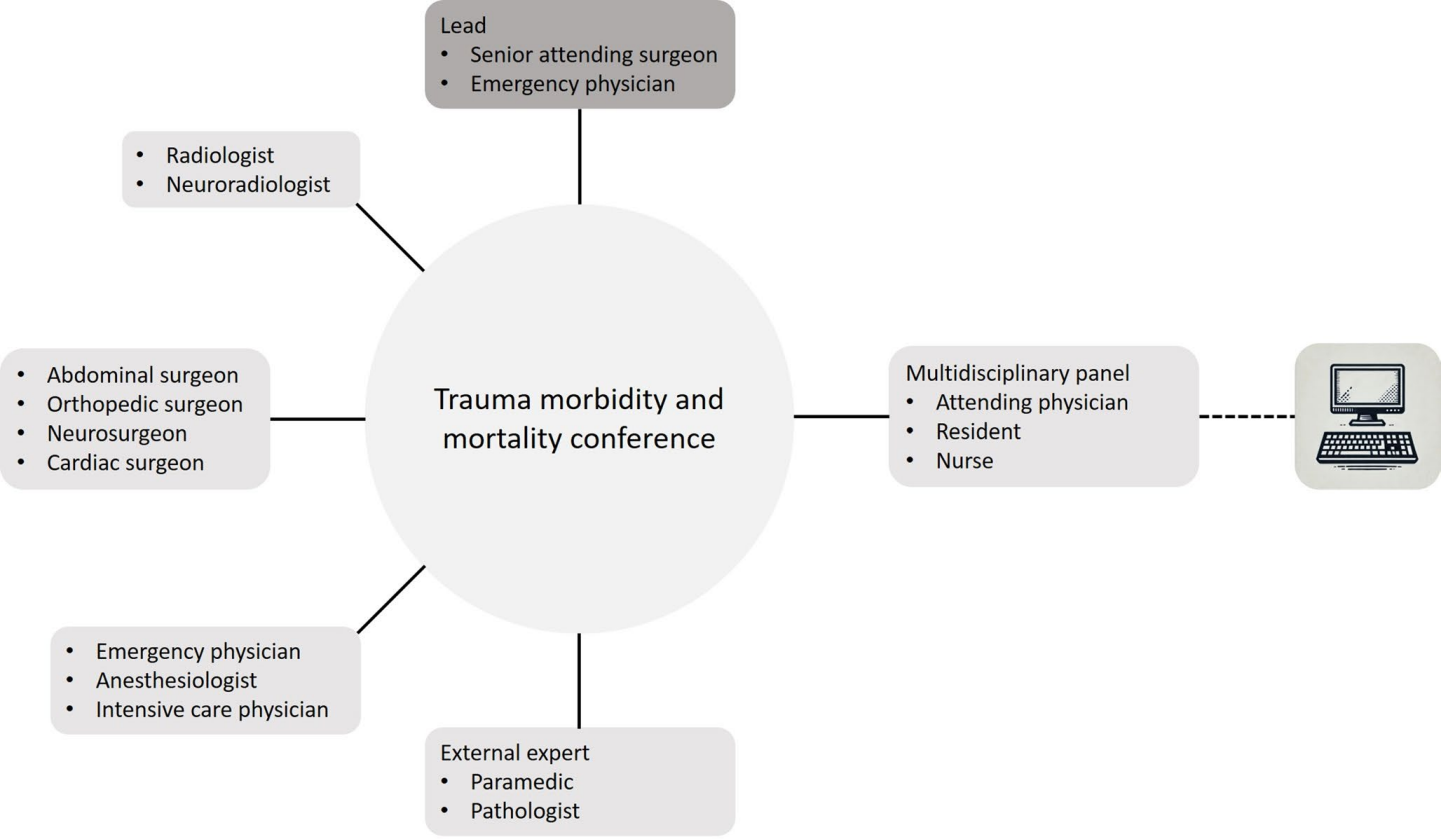
Schematic structure of a trauma morbidity and mortality conference. The schematic computer symbolizes the hybrid nature of the conference with a meeting on-site or via the internet

Since 2013, a monthly Trauma M&M has been held (Fig. 1). A senior attending surgeon or emergency physician chairs the Trauma M&M, with an emergency physician presenting trauma patients according a standardized format. A senior radiologist presents relevant imaging in a chronological accurate way. The multidisciplinary panel, including attending and resident physicians, nurses, and caregivers from various specialties (abdominal, orthopedic, and neurosurgery, radiology, intensive care, anesthesiology, and emergency medicine), critically discuss potential shortcomings such as communication failures, knowledge and skill gaps or infrastructural limitations. Patient characteristics, trauma details, timelines, and the course of care in the emergency department, operating room, intensive care unit and ward are documented in a standardized manner, including any identified potential errors.

### Error categorization

At the Trauma M&M, the identification of potential errors was made by the multidisciplinary plenum by consensus. This was based on the error definition as “the failure of a planned action to achieve its desired goal” [16]. All potential errors were categorized according following specifically developed system. More than one error may have occurred per patient. Subsequently, errors were stratified according on whether they occurred because of a previous problem or not. For example, if a delay resulted from miscommunication, both, the miscommunication as well as the delay accounted as two potential errors.

1. *Communication error*: Failures or gaps in communication between trauma team members. Thereby a communication error violates one of the four communication-maxims of quality, quantity, relevance and manner [17].
2. *Skill or knowledge errors*: Complications and adverse events in procedures performed by trauma team members related to the lack of sufficient skill or necessary knowledge (e.g. misplacement of chest tubes or volume overload with crystalloids).
3. *Logistic errors*: Lacking or misuse of infrastructural resources (e.g. unavailable operating room capacity).
4. *Device errors*: Problems that are primarily caused by malfunction of a medical device.
5. *Delay of a diagnostic or therapeutic procedure*: Relevant delay of necessary diagnostic or therapeutic measures (e.g. CT scan, laparotomy).
6. *Delay of specialized staff*: Relevant delay of specialized personnel (e.g. a surgeon who arrives too late for an emergency department thoracotomy). The exact definition of the timely manner is determined by the Trauma M&M plenum.
7. *Missed injuries*: Injuries resulting from trauma that were not detected during the initial or secondary surveys, including CT scans, however, identified later, including at an autopsy.
8. *Deviation from clinical algorithm*: Deviations from diagnostic or therapeutic algorithms (e.g. ATLS^®^) as well as specific in-house algorithms.
9. *Triage errors*: Incorrect classification in the 3-stage triage system (A-, B- or C-Trauma Team activation) as well as choice of premises and inherent monitoring opportunities. Undertriage was defined as assigning a lower trauma team level than required based on patient condition (e.g. Team B instead of Team C). Accordingly, overtriage refers to the incorrect assignment of a higher triage acuity level than needed.

### Ethics statement

This study has been approved by the Ethics Committee of Canton Bern (EK Bern 2023 − 01875) and was deemed to meet the requirements of the Swiss Human Research Act (Humanforschungsgesetz, HFG) without concerns. An exemption from requiring formal ethics approval was granted, as no methodological outcome associations were performed in this descriptive study, and no biological material was used. Nevertheless, the study adhered to the 1964 Helsinki Declaration, including its later amendments, and upheld comparable ethical standards.

## Results

A total of 198 trauma patients were discussed during the 10 years of Trauma M&M. Nine patients were excluded because of incomplete data, resulting in a total of 189 patients that were further analyzed.

### Demographics

Demographics and trauma-specific data for the study population are summarized in Table 1. Among the 173 patients (92%) who suffered from blunt trauma, the most frequent mechanisms of injury were falls from heights (48%) and vehicle collision (37%). Overall, the median ISS was 32 (IQR 25–43). There were eight patients having an ISS < 16 points. Severe injuries (Abbreviated Injury Score [AIS] ≥ 3) included following body regions: head (*n* = 124), chest (*n* = 78), lower extremity (*n* = 46), and abdomen (*n* = 40). Regarding the admission time, it was found, that 66 patients (35%) were admitted during the morning shift (7 a.m. – 3 p.m.), 98 (52%) during the evening shift (3 p.m. – 11 p.m.), and 25 (13%) during the night shift (11 p.m.– 6 a.m.). Table 2 is summarizing the acute therapeutic interventions of the study population. The most common interventions included emergency laparotomies (*n* = 26), decompressive craniectomies (*n* = 19), and emergency department resuscitative thoracotomies (EDT) (*n* = 13).

**Table 1:** Demographics of patients, discussed at the trauma morbidity and mortality conferences

Variable	Total (*n* = 189)
Male sex, *n* (%)	128 (68)
Age, median years (IQR)	59 (37–76.5)
Blunt trauma, *n* (%)	173 (92)
ISS, median (IQR)	32 (25–43)
Overall 90-day mortality, *n* (%)	145 (77)
Time to death, median hours (IQR)	13 (1–48)
Hospital length of stay*, median days (IQR)	38 (20–96)
Trauma mechanism	
Fall higher than 2 m, *n* (%)	69 (37)
Fall less than 2 m, *n* (%)	21 (11)
Vehicle collision, *n* (%)	69 (37)
Gunshot, *n* (%)	11 (6)
Stabbing, *n* (%)	4 (2)
Strangulation, *n* (%)	2 (1)
Other cause, *n* (%)	13 (7)

*ISS* Injury Severity Scale. *Calculated from all patients without 90-day mortality

**Table 2:** Number of the performed interventions

Intervention	Total (*n* = 92)
Laparotomy	26
Decompressive Craniectomy	19
Emergency department Thoracotomy	13
REBOA	12
ECMO	7

*REBOA* resuscitative endovascular balloon occlusions of the aorta; *ECMO* extracorporal membrane oxygenation

Of the 189 patients analyzed here, 145 (77%) died within 90 days. Among the fatalities, 114 patients (78%) died in the emergency department (ED), and 31 (22%) succumbed in the further follow-up. Of the 145 deaths, 14 (10%) resulted in following organ donations: eight hearts, five lungs, six pancreases, 11 livers and 24 kidneys. The median time to death was 13 h (IQR 1–48), with 37% of deaths occurring within the first three hours after ED admission.

### Analysis of potential errors

**Fig. 2:**
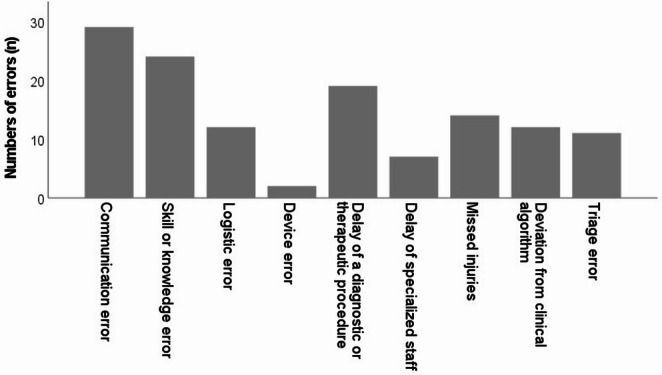
Incidence of categorized errors

In the 189 patients analyzed, a total of 130 potential errors (78 potential errors in 145 fatalities and 52 potential errors in 44 non-fatalities) were identified at the Trauma M&M. Figure 2 highlights the characteristics of these potential errors. The most frequent errors were communication errors (*n* = 29), lack of skill or knowledge (*n* = 24) and procedural delays (*n* = 19). Among the delays, there were 14 diagnostic and five therapeutic delays. Device failures were the rarest type of errors, occurring in two patients. In one case, there was an incorrect measurement of the hemoglobin level with a point-of-care testing device and in the other case, an extracorporeal membrane oxygenation (ECMO) device had to be replaced due to a malfunction. Overall, 12 deviations from established algorithms were identified and classified as errors. These deviations primarily involved protocols related to EDT, level of triage, trauma team activation, ATLS^®^-based primary surveys, chest tube insertion and X-rays performed as part of the primary survey. Eleven patients were classified as under-triaged, with no cases of over-triage identified.

Focusing on interventions, EDT (*n* = 13) was most susceptible for potential errors. These included chaotic team communication due to loud, incomprehensible conversation during the procedure, unclear responsibilities combined with an inefficient team lead. Additionally, there was a consecutive failure to insert a chest tube, the impairment of a concomitant laparotomy, and a missed time-out prior to EDT. Furthermore, EDT was not performed in five patients due to a lack of available skilled personnel. Resuscitative endovascular balloon occlusion of the aorta (REBOA) was applied in 12 patients, with five associated errors. These errors included prolonged application, malposition (too distal and extravascular) in two cases, balloon inflation with air, and repetitive failure of arterial sheath insertion.

### Subsequent errors to communication errors

**Fig. 3:**
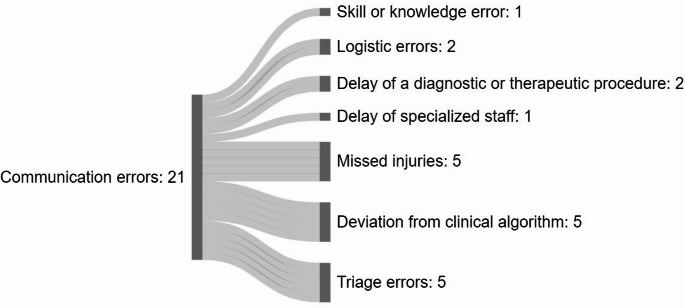
Sankey diagram illustrating the flow of communication errors to subsequent sequential errors

Of the 29 communication failures, 21 (72%) led to subsequent errors in various categories, as shown in Fig. 3. Missed diagnoses and under-triage were frequently associated with miscommunication between medical teams. In several cases, crucial findings were either not conveyed or not emphasized sufficiently, leading to delayed or missed diagnoses. These included an undiagnosed diaphragmatic rupture due to unclear reporting of imaging findings and a failure to communicate the location of penetrating trauma to the abdominal surgeon. Similarly, a tibial fracture was overlooked because an uncompleted secondary survey was not mentioned during patient handover. In another case, a suspected sternal fracture was overlooked, because the trauma mechanism and clinical suspicion were not communicated to radiology, preventing the acquisition of detailed reconstructive imaging. Additionally, a mesenteric laceration went undetected due to the lack of communication regarding the high-energy character of a blunt trauma. Lastly an esophageal laceration was missed because radiology failed to communicate its recommendation for an ECG-synchronized CT scan to the trauma team leader.

Deviations from standard treatment algorithms also emerged as a major concern (*n* = 5) driven by communication breakdowns within the interdisciplinary team (Fig. 3). In one case, a pneumothorax was not detected prior to CT scan because a critical team time-out was omitted after primary survey. In another case, a patient with severe traumatic brain injury and concomitant splenic injury primarily underwent splenic artery embolization resulting in a potential delay to neurosurgery. Here, a simultaneous splenectomy and neurosurgical intervention was judged by the Trauma M&M to be the better approach. Similarly, a patient was transferred before completing the secondary survey, as the team failed to acknowledge that essential assessments were still pending. Additionally, the decision-making process was disrupted in a case where a CT scan was performed prior to necessary intubation. This error was compounded by uncertainty regarding the patient’s living will and a lack of leadership in clarifying the appropriate course of action.

### Reevaluation of clinical algorithms

In 43 incidences, several clinical algorithms were discussed, reevaluated, revised, or confirmed during Trauma M&M. These discussions led to revised shockroom guidelines including adjustments of triage protocols. Moreover, algorithms for the indications for EDT and REBOA including cardiopulmonary resuscitation after penetrating trauma were created.

## Discussion

Severely injured patients are particularly susceptible to diagnostic and therapeutic errors due to the urgency and time-sensitiveness of decisions and uncertainties regarding medical history and trauma mechanisms [18]. In this retrospective case series of 189 severely injured trauma patients, a specific categorization of potential errors has been created based on the findings of the monthly Trauma M&M held at a Swiss Level I Trauma Center. It was found, that communication errors emerged as the most common type of errors. This was followed by skill or knowledge gaps and the delay to diagnostic or therapeutic procedures. Moreover, it was found, that communication errors, specifically, resulted in subsequent errors including missed diagnoses, under-triage or deviation from clinical algorithm.

An essential approach to improving care for trauma patients is the identification of potential errors through Trauma M&M conferences, with the aim of incorporating the “lessons learned” into the daily clinical practice and training [18]. Previous studies on trauma care errors have focused predominantly on preventable or potentially preventable deaths. It was found, that insufficient hemorrhage control and airway management being the leading contributors to mortality [5–7, 18, 19]. However, these studies have not addressed the underlying contributors in detail, such as communication errors, that lead to these outcomes. Evidence suggests that communication failures significantly contribute to errors and require targeted interventions [20, 21]. In the current study, communication errors resulted in missed or delayed diagnoses, under-triage, and deviations from clinical algorithms. These findings, visualized in Fig. 3, underline the significant role of precise communication in a “chain of potential errors”. Recognizing the cascade effect of miscommunication, we have implemented structured handoff protocols, time-outs, and targeted communication training as core strategies to mitigate these errors. To our knowledge, this is the first study to specifically analyze these sequential errors within a trauma population. Further prospective research is warranted to understand the ripple effects of communication problems across various trauma systems, as this has the potential to improve care across all error categories, even in well-established trauma systems [22, 23]. Further, the emergence of EDT and REBOA as a critical error-prone procedure in this study, highlighting the need for intensive training focused on both communication and technical aspects. This finding underscores on one hand the broader need for communication training within trauma teams, as communication problems often underpin other errors. On the other hand, persistent algorithms can be adjusted or implemented to ease daily clinical practice. Beyond serving as a forum for reflective practice, the Trauma M&M process functions as a continuous quality control mechanism. Clinical algorithms are regularly reviewed and directly revised when systematic issues are identified through case analysis. These modifications are incorporated into updated standard operating procedures (SOPs) and implemented in practice. Adherence to these updates is monitored through structured follow-up and internal audits, ensuring that insights gained from the Trauma M&M process translate into sustained improvements in trauma care. It is important to emphasize, that the aim of the Trauma M&M is not to point out the mistakes of individuals, but to highlight the treatment quality of the entire Trauma team. Of note, the Trauma M&M has no medico-legal consequences. Independently to the Trauma M&M, all employees of Bern University Hospital have the opportunity to report incidences anonymously within the local critical incidence reporting system (CIRS).

A standardized error categorization system, as proposed here, is vital for advancing research in this area. This categorization aligns with methodologies used in previous studies while omitting less feasible aspects, such as internal processing classifications [5–7, 18]. Using this framework, future research can delve deeper into the specific effects of communication problems and develop tailored solutions for different contexts. While J. Reason’s widely accepted definition of error was applied in this study, Grice’s communication model was uniquely used to analyze communication errors retrospectively [16, 17]. Although effective, the use of this model may introduce measurement uncertainties and limitations in validity.

Trauma M&M conferences are indispensable for the education and training of medical professionals, particularly residents and new staff. These forums provide a collaborative environment for learning, sharing best practices, and addressing complex trauma cases, such as penetrating trauma, which are less common in certain regions [15]. This study highlights the importance of interdisciplinary discussions in Trauma M&M, fostering collaboration and developing a common language among various specialties, including emergency medicine, surgery, anesthesiology, radiology, and intensive care. The consistent implementation of these meetings over a decade has not only improved the quality of care but also strengthened interprofessional understanding and teamwork. Structured reviews of cases during Trauma M&M allow for the identification of learning objectives, which can then be directly addressed. The structured nature of the Trauma M&M, combined with regular attendance documentation has been recognized by the Swiss Society for Surgery, the Swiss Orthopedics, the Swiss Society of Radiology, the Swiss Society of Emergency Medicine, and the Swiss Society of Anesthesiology with continuing education credits.

This study has limitations. The current study population is a selection of trauma victims treated at Bern University Hospital that were discussed at the Trauma M&M. Due to this selection bias, it is therefore not possible to calculate the overall mortality or complication rate of all trauma patients at Bern University Hospital. Moreover, as a single-center study from a well-resourced Swiss Level I trauma center, generalizability to other high-volume or resource-limited centers may be limited. Differences in trauma burden, staffing, and infrastructure could influence both error patterns and the impact of Trauma M&M interventions. Deaths are consistently reviewed, additional cases are nominated by any caregiver, encouraging multidisciplinary participation. To reduce underreporting, we foster a non-punitive culture that emphasizes learning over blame. Regular reminders from department leadership further encourage open reporting. While the inclusion of cases without rigid criteria, apart from trauma as the primary reason for admission, broadens the spectrum of discussed patients, this study has a selection bias occurring from the inclusion through expert opinions. On the other hand, this approach addresses the known limitation of focusing solely on preventable deaths, which represent only the “tip of the iceberg” [5–7, 18]. The broad and often hidden base of the iceberg, includes non-fatal but impactful events as addressed in this study (e.g. communication failures that contribute to a cascade of subsequent errors, including delays, missed injuries etc.). However, the low autopsy rates available for Trauma M&M in Switzerland present challenges in reliably assessing the impact of errors on outcomes, even in fatal cases. At present, Switzerland lacks a supra-regional trauma error registry. Our local Trauma M&M serves as a forum to discuss the performance of the Trauma Team in selected cases, however, does not serve as a complete documentation system of all potential errors.

The prominence of communication problems in this study underscores the need for concurrent and anticipatory teaching, which is facilitated by the Trauma M&M format. Hybrid participation models, incorporating both in-person and web based attendance, could enhance engagement, especially from lower-level trauma care centers [24, 25]. Additionally, the Trauma M&M setting provides a unique platform for discussing algorithm deviations and ensuring that adjustments are made judiciously, preventing unnecessary overcorrections. Moreover, Trauma M&M conferences play a pivotal role in promoting quality, safety, and transparency in trauma care [26]. Ongoing peer review of error patterns and Trauma M&M designs is essential for future advancements, particularly given regional variations in epidemiology, injury patterns, and trauma systems.

## Conclusion

The review and analysis of the standardized Trauma M&M protocols of 189 patients revealed communication problems as the leading cause for errors followed by skill/knowledge gaps as well as delays in diagnostic and treatment measures. This reveals the need for communication problems to be a self-contained category for Trauma M&M with a high potential for further improvement. It further shows the importance of continuous communication training for trauma teams. More research with comparable classification is needed to gain a better understanding of error patterns based on communication problems. Ultimately, the analysis of ten years of Trauma M&M in a Swiss Level I trauma center underlines its important role for the continuous improvement, interdisciplinary collaboration and education and its role in improving everyday clinical practice e.g. through revision or creation of trauma relevant treatment algorithms.

## Data Availability

No datasets were generated or analysed during the current study.
